# Supplementary material for: Characterisation and modelling of potassium-ion batteries
Source: Nat Commun. 2024 Aug 31;15:7580. doi: 10.1038/s41467-024-51537-w (PMC11365988; doi:10.1038/s41467-024-51537-w)
Supplement: Supplementary file 2 — Supplementary Information [file 41467_2024_51537_MOESM2_ESM.pdf]

Supplementary Information

Characterisation and Modelling of Potassium-Ion Batteries

Shobhan Dhir[1], John Cattermull[1,2], Ben Jagger[1], Maximilian Schart[1], Lorenz F. Olbrich[1], Yifan Chen[1], Junyi Zhao[1], Krishnakanth Sada[1], Andrew Goodwin[2], and Mauro Pasta[1*]

[1]Department of Materials, University of Oxford, OX1 3PH, UK
[2]Inorganic Chemistry Laboratory, Department of Chemistry, University of Oxford, OX1 3QR, UK
[*]Corresponding author: mauro.pasta@materials.ox.ac.uk

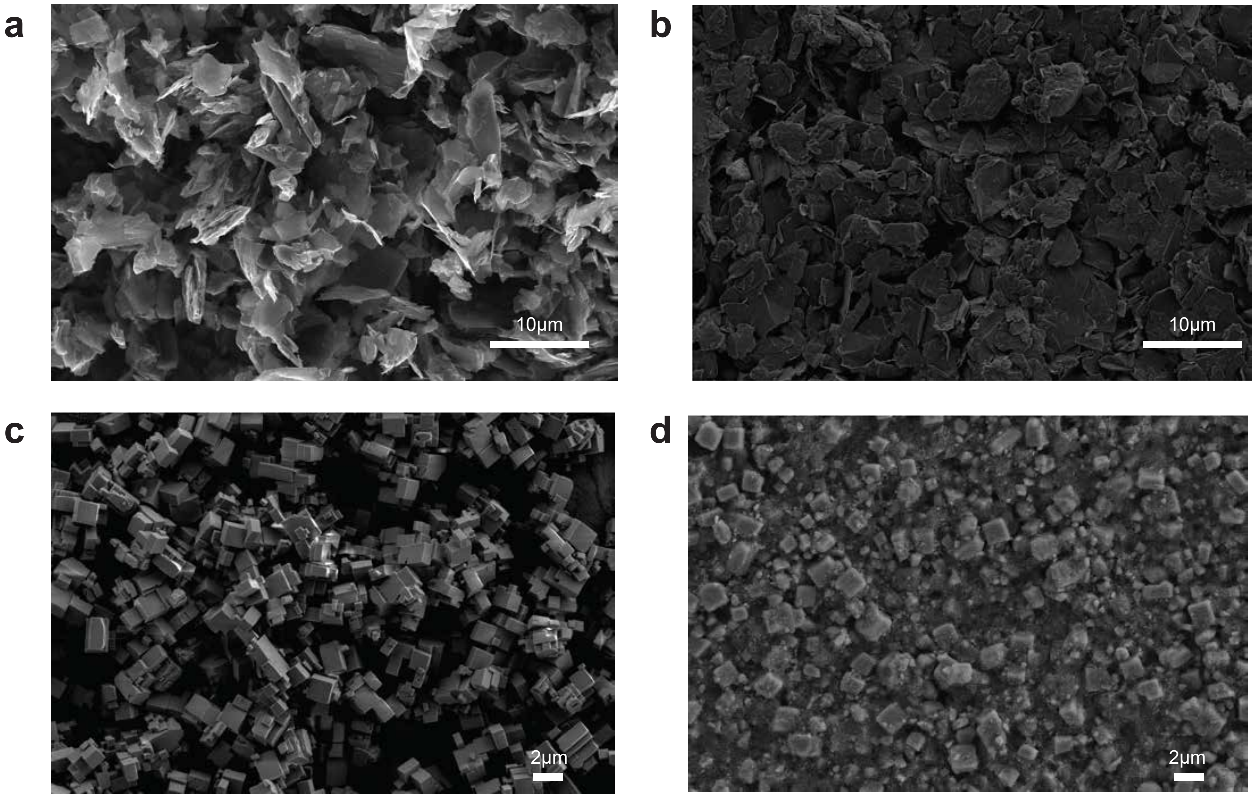

Supplementary Fig. 1 Additional SEM images. (a) Pristine graphite and (b) Cast graphite electrode (c) Pristine KMF material (d) Cast KMF electrode

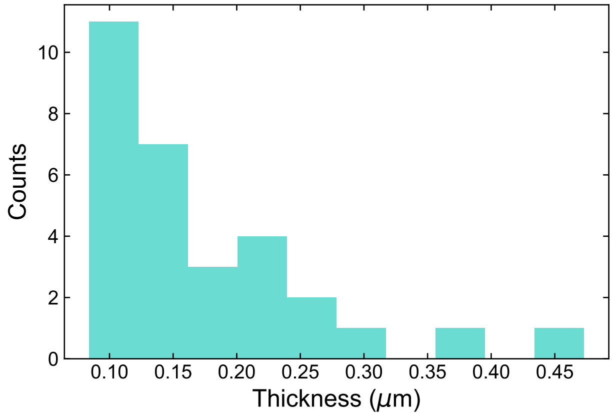

Supplementary Fig. 2 Graphite particle thicknesses from SEM analysis

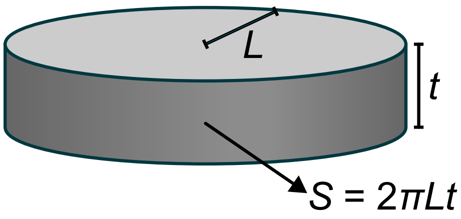

Supplementary Fig. 3 Disc used to model graphite particles. Shaded edge area represents the active area for intercalation

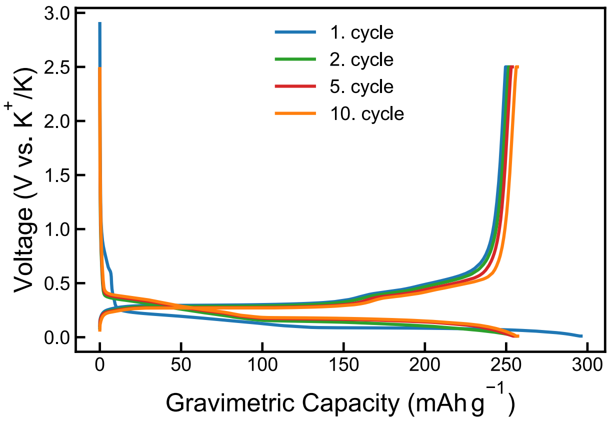

Supplementary Fig. 4 Galvanostatic cycling at C/20 of graphite half-cell in 2 m KFSI TEP at 30°C

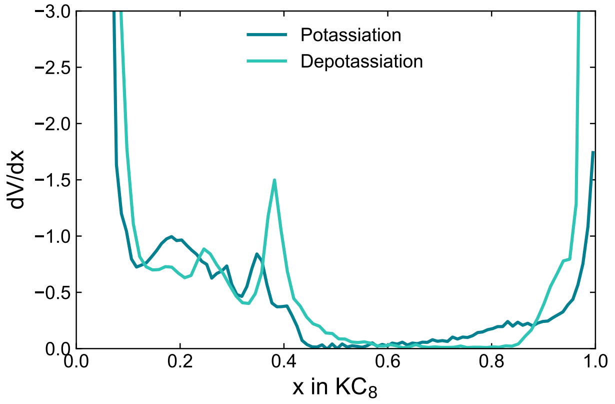

Supplementary Fig. 5 Kang-Chueh GITT graphite derivative of the Nernst potential $(\frac{\partial V_{eq}}{\partial x})$ over stoichiometry at 20°C

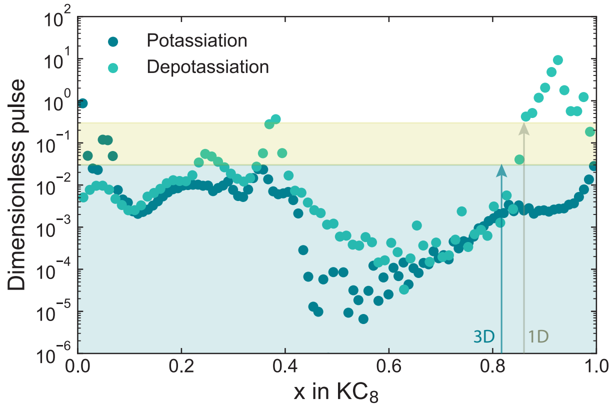

Supplementary Fig. 6 Example dimensionless pulse $\hat{\tau}$ for graphite electrode over stoichiometry from Kang-Chueh GITT in three-electrode cell at 20°C. Coloured region indicates the ideal valid range for the semi-infinite assumption in planar (1D) and spherical (3D) geometries[1]

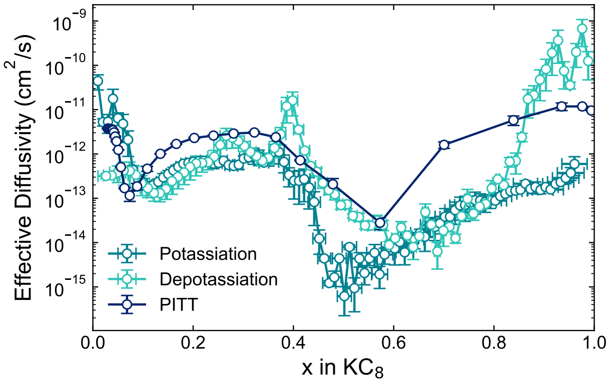

Supplementary Fig. 7 Long-time PITT compared to GITT effective diffusivity for graphite at 20°C

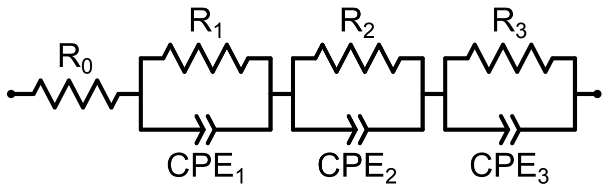

Supplementary Fig. 8 Equivalent circuit used to fit graphite impedance data, as reported by O'Regan et al.,[2] with an additional R||CPE element included to fit the low frequency tail

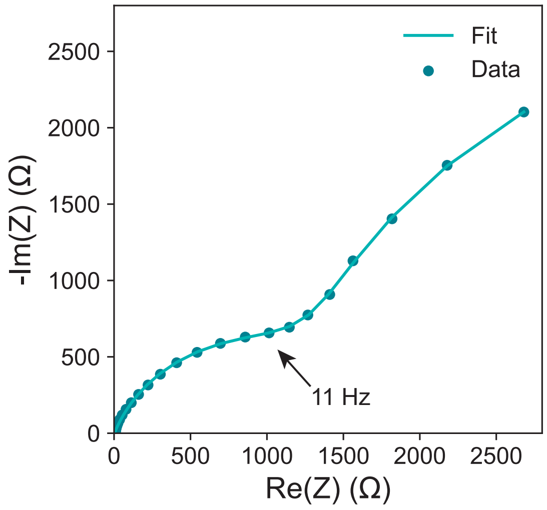

Supplementary Fig. 9 Example K graphite impedance spectra in three-electrode cell > 0.5 Hz at 20°C

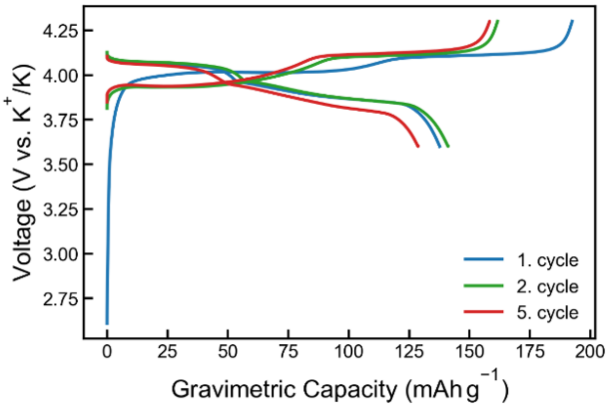

Supplementary Fig. 10 Galvanostatic cycling at C/20 of KMF half-cell in 2 m KFSI TEP at 30°C

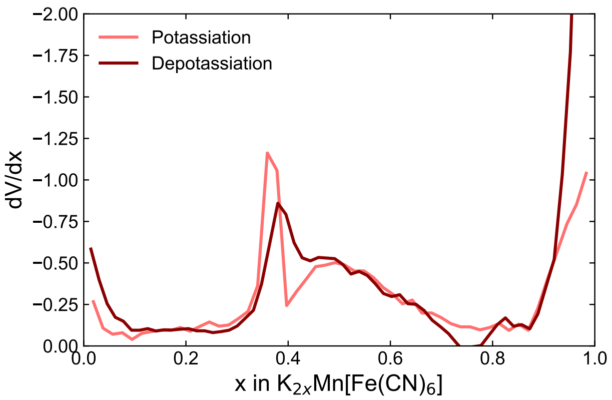

Supplementary Fig. 11 Kang-Chueh GITT KMF derivative of the Nernst potential ($\frac{\partial V_{eq}}{\partial x}$) over stoichiometry at 20°C

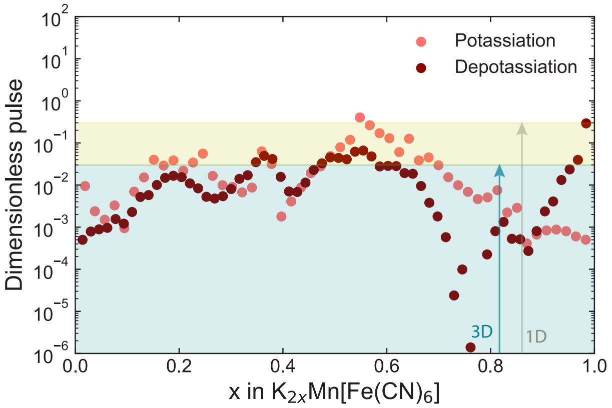

Supplementary Fig. 12 Example dimensionless pulse $\hat{\tau}$ for KMF electrode over stoichiometry from Kang-Chueh GITT in three-electrode cell at 20°C. Coloured region indicates the ideal valid range for the semi-infinite assumption in planar (1D) and spherical (3D) geometries[1]

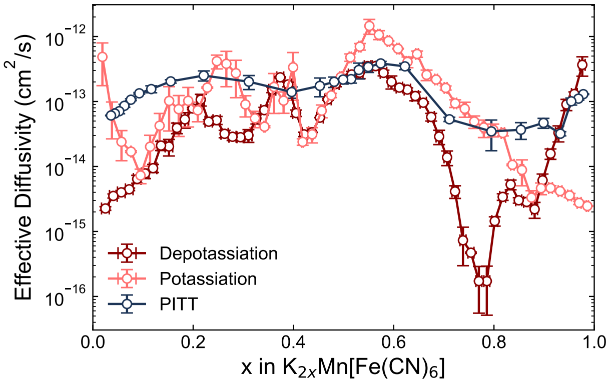

Supplementary Fig. 13 Long-time PITT compared to GITT effective diffusivity for KMF

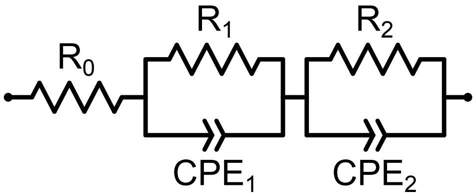

Supplementary Fig. 14 Equivalent circuit used to fit KMF impedance data, as reported by O'Regan et al.[2]

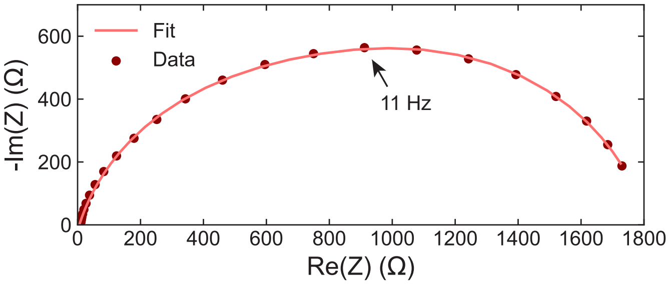

Supplementary Fig. 15 Example KMF impedance spectra in three-electrode cell at 20°C

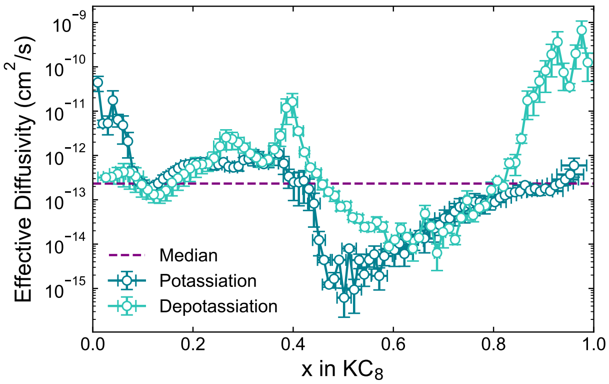

Supplementary Fig. 16 Median graphite diffusivity used in the modelling

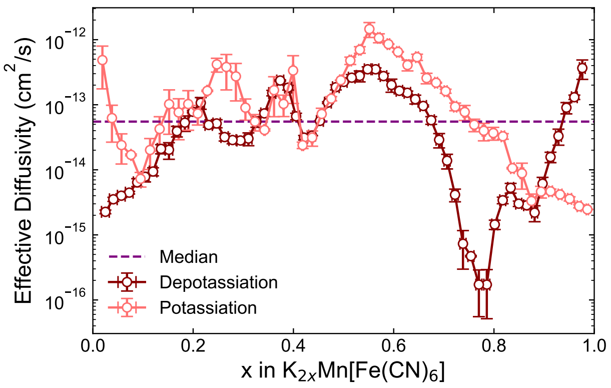

Supplementary Fig. 17 Median KMF diffusivity used in the modelling

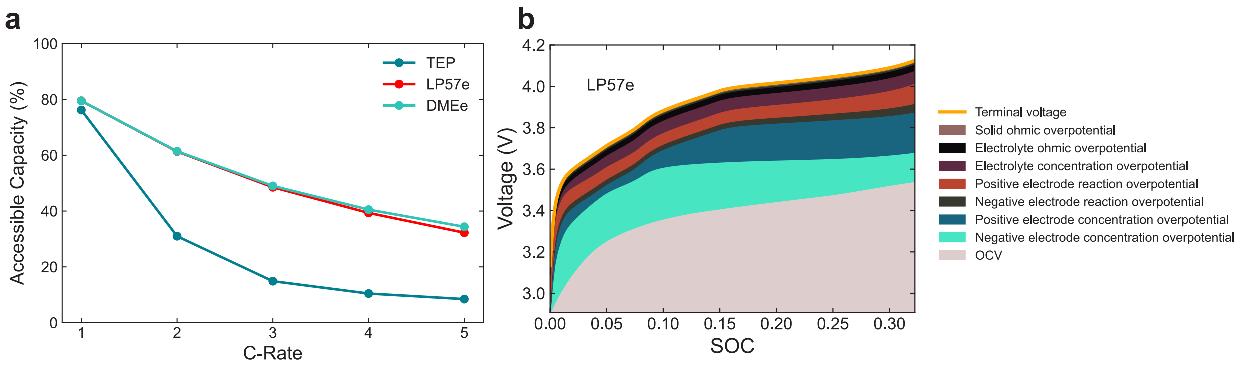

Supplementary Fig. 18 Full-cell Doyle-Fuller-Newman (DFN) simulations of three K-ion cells with different electrolytes in a cell based on the commercial LG M50 cylindrical cell format. The state-of-the-art modelled K-ion cell is $K_2Mn[Fe(CN)_6]$ (KMF) | | graphite (G) with 2m KFSI TEP electrolyte (denoted as TEP). One cell is a hypothetical electrolyte case using the characterised electrolyte properties of the 2m KFSI:DME[3] (DMEe). The final cell is a hypothetical electrolyte case using the characterised electrolyte properties of the Li-ion electrolyte 1M LP57[4] to simulate K-ion performance with a LP57 equivalent (LP57e) electrolyte. The cell energy is 7.3 Wh. NP ratios were kept constant at 1.1 with constant electrode porosities, and properties for all chemistries. Modelled using PyBaMM[5] at 20°C (**a**) Accessible capacities at increasing C-rate (**b**) LP57e cell overpotential components during a 5C charge

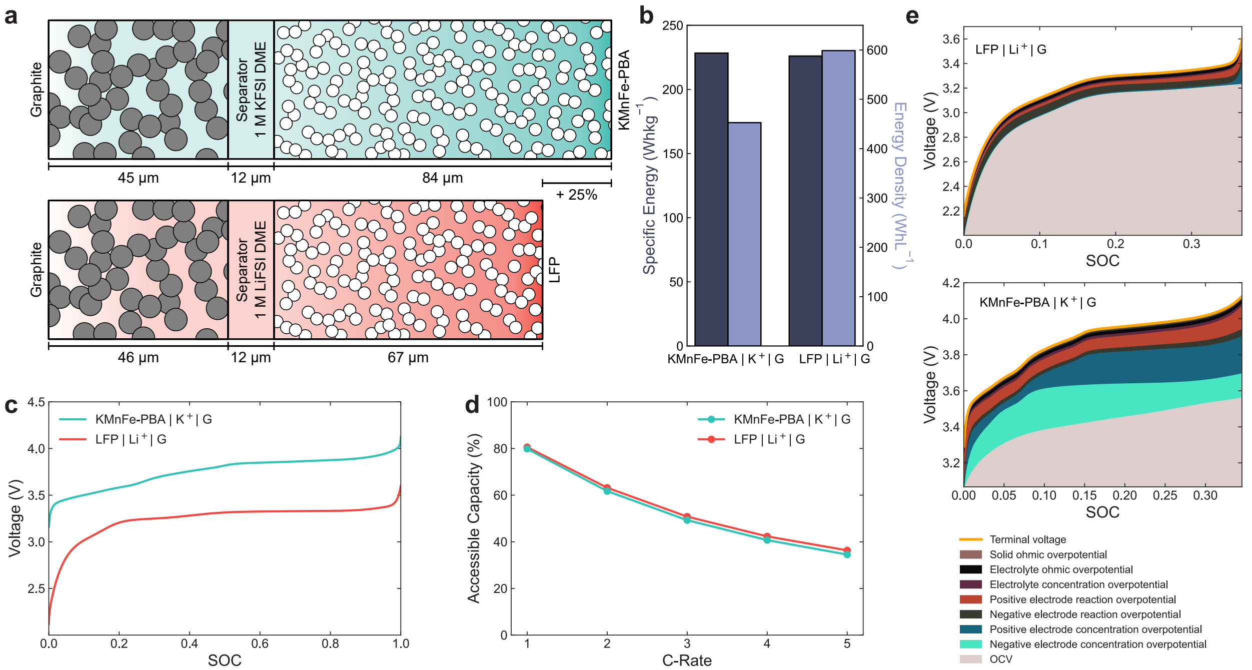

Supplementary Fig. 19 K-ion and LFP Li-ion full cell simulations Full-cell Doyle-Fuller-Newman (DFN) simulations of K-ion and LFP Li-ion cells in a cell based on the commercial LG M50 cylindrical cell format. The modelled K-ion and Li-ion cells are $K_2Mn[Fe(CN)_6]$ (KMF) | 1 M KFSI DME | graphite (G) and $LiFePO_4$ (LFP) | 1 M LiFSI DME | graphite (G), respectively. The composition-dependent electrolyte properties are from.[3] The cells are matched in energy at 7.3Wh by adjusting electrode thicknesses. NP ratios were kept constant at 1.1 with constant electrode porosities for both chemistries. Modelled using PyBaMM.[5] The LFP material parameters were taken from the PyBaMM 'Prada2013' parameter set.[6,7] LFP particle size was taken from BatPaC 5.1 and used for the cathode particle size for both chemistries.[8] (**a**) Schematic of simulated cell geometries (**b**) Energy density and specific energy of the two chemistries at the stack level using the stack-level model developed previously[9,10] (**c**) Simulated galvanostatic profiles for the K-ion and Li-ion cells (**d**) Accessible capacities at increasing C-rate (**e**) Overpotential components during a 5C charge (1C = 1.91 mA cm^{-2} and 2.24 mA cm^{-2} for the for the K-ion and LFP Li-ion cells, respectively)

1 Supplementary Note 1: PITT

For an additional characterisation and comparison PITT was also utilised to characterise $\widetilde{D}_{\mathrm{g}}$ and $\widetilde{D}_{\mathrm{KMF}}$. For the long-time PITT analysis,[11,12] which is less susceptible to short-term reaction kinetic overpotential errors, the diffusion length term, L is required. To determine the graphite L (L_g), again from the approximated disc geometry (Supplementary Fig. 3), L_g was taken to be the the disc radius using the average particle size (Fig. 2c). For L_{KMF} from the average particle size (Fig. 3c) and cuboid geometry an equivalent volume sphere was modelled with L_{KMF} being its radius. PITT cannot obtain the high resolution composition dependence possible using GITT in the two-phase regions which is one of the major limitations of PITT, that an insufficiently small potential step can be applied in order to produce a measurable current response, and therefore, the composition change can be large during a single potential step.[13,14] Markevich et al. also showed in a comparison of GITT vs. PITT that PITT was less accurate as it is more susceptible to parasitic current contribution than GITT,[13] also finding the PITT $\widetilde{D}$ results were overestimated and particularly the $\widetilde{D}_{\mathrm{KMF}}$ minima were shallower than those for GITT matching the findings for $\widetilde{D}_{\mathrm{g}}$ and $\widetilde{D}_{\mathrm{KMF}}$ values for PITT here. They concluded GITT is superior particularly when phase transitions are involved[13,14] as in this case. Since there can also be no zero-current current response transient in PITT the current-related overpotential errors are inherent therefore, the Kang-Chueh GITT method has an inherent advantage there also.[1]

2 Supplementary Note 2: Synchrotron X-ray diffraction

2.1 Measurements

Our powder synchrotron X-ray diffraction data were collected on the I11 beamline at Diamond Light Source, UK. The diffraction patterns of the sample were collected in capillary

transmission geometry. A room temperature X-ray diffraction pattern was collected using the Mythen2 Position Sensitive Detector (PSD), two data collections of 20 seconds each were taken at angles 0.25 degrees apart, then summed to account for gaps in the detector coverage.

2.2 Refinement details

The structural refinement for $K_2Mn[Fe(CN)_6]$ was performed using a Rietveld refinement in TOPAS software,[15] in conjunction with distortion modes obtained from ISODISTORT.[16,17] The parent cell was a cubic crystal structure of $K_2Mn[Fe(CN)_6]$ in $Fm\bar{3}m$ taken from the undistorted cubic cell,[18] with crystallographic details given in Table 1. K occupancies were allowed to refine freely and refinement returned an occupancy of 1.871(3). The uncertainties reported from the refinement in TOPAS are likely overly precise, hence why ICP-MS was employed to back-up the evidence.

Positions of all of the K, C, and N atoms were allowed to refine as a function of the 21 distortion modes generated by a distortion from $Fm\bar{3}m$ to $P2_1/n$, many of which were minor in contribution, but allowed by symmetry. In order to reduce the number of free parameters, the thermal displacement parameters for transition metals Mn and Fe were constrained to be equal; so too for those of the C and N sites. The K site was allowed to refine with an independent thermal displacement parameter. Finally, an anisotropic peakshape model for a monoclinic unit cell was applied to account for the different strains in different directions that individual crystallites will have, giving a better interpretation of the peak intensities.[19]

For the graphite sample, we performed a Pawley refinement on the diffraction pattern which was collected primarily to confirm the crystallinity. The inevitable problem of preferred orientation of crystallites meant Rietveld refinement would not provide tangible insight into the structure. Lattice parameters of $a = 2.46073(5)$ Åand $c = 6.71841(5)$ Åwere extracted from the refinement in $P6_3/mmc$, agreeing with literature values.[20]

Supplementary Table 1 Crystallographic details for the $Fm\bar{3}m$ parent cubic structure[18]

a(Å)	10.11349		
Z	4		
Atom	x	y	z
C	0.1821	0.0	0.0
Fe	0.0	0.0	0.0
K	0.25	0.25	0.25
Mn	0.5	0.0	0.0
N	0.289	0.0	0.0

Supplementary Table 2 Crystallographic parameters for $P2_1/n$ structure of $K_{1.871(3)}Mn[Fe(CN)_6]$ at room temperature from the fit to the data

a (Å)	10.107(4)			
b (Å)	7.3162(10)			
c (Å)	6.9774(10)			
β (°)	89.9327(10)			
V (Å^3)	515.9(2)			
Z	4			
Atom	x	y	z	U_{iso} (Å^2)
C1	-0.0369(10)	0.8458(15)	0.7673(15)	0.0129(8)
C2	-0.0390(10)	0.2155(15)	0.8332(15)	0.0129
C3	0.8155(9)	-0.0087(15)	0.0552(13)	0.0129
Fe	0	0	0	0.0134(3)
K	0.7509(5)	0.56116(15)	-0.0195(3)	0.0396(6)
Mn	0.5	0	0	0.0134
N1	-0.0379(8)	0.7639(12)	0.6391(12)	0.0129
N2	0.9473(8)	0.3404(12)	0.7692(12)	0.0129
N3	0.7065(8)	-0.0252(11)	0.1018(10)	0.0129

3 Supplementary Note 3: Electrolyte characterisation

The 2 m KFSI:TEP electrolyte characterisation has been reported by Zhao et al. and the values are reported in Supplementary Table 3.[21]

The KFSI:DME electrolyte properties were characterised in our previous work and their parameterised forms as a function of either molar (c) or molal concentration (c_m) are implemented here:[3]

$t_+^0 = 0.497 - 0.074c_m$

$$\chi_M = (1 + 0.071c_m)(1 - 1.833{c_m}^{\frac{1}{2}} + 1.608c_m)$$

$$D = 1.003 \times 10^{-9} - (3.104 \times 10^{-10})c_m + (3.633 \times 10^{-11}){c_m}^2$$

$$\kappa = 15.970 \left(\tfrac{c}{1.493}\right)^{1.620} \exp\left(0.023(c - 1.493)^2 - \tfrac{1.620}{1.493}(c - 1.493)\right)$$

$$c = 0.859c_m - 0.051{c_m}^2$$

4 Supplementary Note 4: Modelling - LP57 comparison

As the DMEe electrolyte transport properties are fast, an additional comparison was made with another hypothetical electrolyte, this time using an electrolyte with the properties of the commercial Li-ion electrolyte LP57 fully characterised by Landesfeind *et al.*[4] (Supplementary Fig. 18). 1 M LP57 was used as another benchmark indicating current Li-ion commercial electrolyte performance. Supplementary Fig. 18a shows using this hypothetical equivalent LP57 (LP57e) electrolyte results in very little difference with the DMEe electrolyte at 5C rate (32% vs. 34% accessible capacity, respectively), despite the salt diffusivity and ionic conductivity of 1 M LP57 being $\sim$ half that of 2 m KFSI:DME at 20°C.[3,4] This shows that even if a K-ion electrolyte with the slower transport properties of LP57 is developed, the electrode properties are rate limiting.

5 Supplementary Note 5: Modelling - LFP comparison

Since the LFP LIB is the Li-ion chemistry that K-ion would be competing with,[9] an LFP model in the same LG M50 cylindrical cell was also simulated for comparison. For fair comparison the total energy of the K-ion and LFP cell was matched at 7.3 Wh by adjusting the electrode thicknesses and the particle sizes for each chemistriy's cathode and anode materials kept constant, respectively. KFSI and LiFSI in DME electrolytes were used for the K-ion and LFP Li-ion cell, respectively for additional fair comparison and because both

electrolytes were fully characterised over concentration in the same conditions previously.[3] For the K-ion cell the half-cell OCV profiles for the KMF cathode and graphite anode determined here were implemented (Fig. 2d and 3d). NP ratios of 1.1 and electrode porosities were kept constant for both chemistries. Both the cathode materials particle sizes were kept constant as 500 nm, the commercial LFP particle size from BatPaC 5.1[8] and the same commercial graphite particle sizes utilised for both chemistries (1 µm).

Supplementary Fig. 19a depicts the K-ion and Li-ion LFP full cells being modelled, with their corresponding thicknesses to match the energies of the cells, showing the KMF cathode is 25% thicker than LFP to match the total cell energies of 7.3 Wh. This is primarily due to the low bulk density of the KMF compared to LFP, limiting the energy density of the K-ion cell. Supplementary Fig. 19b compares the specific energy and energy density of the two chemistries at stack level based on their theoretical capacities and simulated galvanostatic profiles (Supplementary Fig. 19c) using the stack-level model developed previously.[9,10] The results show the K-ion chemistry actually displays a comparable specific energy to the LFP chemistry (1% higher) though a significantly lower energy density (24% lower), again showing K-ion is competitive particularly where mass is a more critical constraint than space. Supplementary Fig. 19c displays the DFN simulated galvanostatic profiles during 1C charge of the two full cells, demonstrating the significantly higher operational voltage of the KIB, which enables it to be competitive particularly in specific energy, despite the lower specific capacities of the K-ion anode and cathode materials compared to the LFP cell. Finally, Supplementary Fig. 19d shows the fast charging performance comparison for the K-ion and LFP cell from the DFN simulations, demonstrating the K-ion cell displays superior rate capability, achieving significantly higher accessible capacities at all rates simulated. Even at the fast charging rate of 5C the K-ion cell can access 54% capacity compared to 36% for the LFP Li-ion cell.

Commercial LFP particles need to be nanosized (here $\sim 10\times$ smaller than the graphite

particles) to increase the surface area available for reaction and decrease the diffusion length due to the substantially lower j_0 and D of LFP than Li^+ graphite. This is similar for the K-ion cell with the lower $\widetilde{D}_{\mathrm{KMF}}$ than $\widetilde{D}_{\mathrm{g}}$ and $\sim$ one order of magnitude lower $j_{0,\mathrm{KMF}}$ than $j_{0,\mathrm{g}}$, therefore, the KMF particles must also be nanosized to match the faster kinetics of the graphite anode.

Exploring this further, Supplementary Fig. 19e plots the overpotential components over SOC for both chemistries during a 5C charge until the upper cut-off voltage is reached, at which point metal plating may start to occur resulting in dramatic capacity losses.[22] First, it is clear that the K-ion cell can experience larger overpotentials before reaching the upper cut-off voltage due to the KIB OCV profile with a more gradual increase compared to that of the LFP Li-ion cell. One of the most significant reasons for this is the higher intercalation potential of K^+ compared with Li^+ into graphite ($\sim$ 0.3 V vs. 0.1 V, respectively).[9] For the K-ion cell the electrode concentration overpotentials are the most significant. The negative electrode concentration overpotential is notably higher for the K-ion cell compared to LFP cell and this is partly due to $\widetilde{D}_{\mathrm{g,K^+}}$ being over an order of magnitude lower than $\widetilde{D}_{\mathrm{g,Li^+}}$ in the model. Though, also because from SOC $\sim$ 0.2 the graphite anode in the LFP cell has an almost constant OCV whilst in the plateau region,[12] therefore, the difference between the OCV at the graphite particle surface Li^+ concentration ($U(c^s_{\mathrm{g,Li^+}})$) compared to the OCV at the bulk graphite Li^+ particle concentration ($U(c_{\mathrm{g,Li^+}})$) is low, minimising graphite concentration overpotentials. Whereas in the K-ion cell the graphite OCV is changing to a much greater extent (Fig. 2d), driving a larger difference between the surface and bulk K^+ OCVs. Further work should also reanalyse $\widetilde{D}_{\mathrm{g,Li^+}}$ using the Kang-Chueh GITT approach to increase its accuracy. Despite the similar $\widetilde{D}_{\mathrm{KMF}}$ and $\widetilde{D}_{\mathrm{LFP}}$, the positive electrode concentration overpotential is larger for the K-ion cell. This is again due to the almost constant OCV for the LFP cathode whilst in the plateau region, again resulting in a very small difference between $U(c_{\mathrm{LFP,Li^+}})$ and $U(c^s_{\mathrm{LFP,Li^+}})$ with increasing SOC compared

to that between $U(c_{\mathrm{KMF,K^+}})$ and $U(c^s_{\mathrm{KMF,K^+}})$, again reducing concentration overpotentials in the LFP cell. For the K-ion cell initially the graphite concentration overpotential is most significant, yet, after SOC $\sim$ 0.25 the KMF concentration overpotential becomes more dominant reflecting the KMF leaving the two-phase OCV plateau between $x \sim 0.95$ to 0.75 (Fig. 3d), reaching the apparently undistorted phase at $0.4 < x < 0.75$ and thus the OCV increasing more rapidly, therefore with decreasing KMF K^+ concentration $\eta_{c,\mathrm{KMF}} = U(c_{\mathrm{KMF,K^+}}) - U(c^s_{\mathrm{KMF,K^+}})$ is larger than $\eta_{c,\mathrm{g}} = U(c_{\mathrm{g,K^+}}) - U(c^s_{\mathrm{g,K^+}})$.

For the K-ion cell both the negative and positive electrode reaction overpotentials are relatively similar in magnitude throughout the charge whereas for the LFP cell the negative reaction overpotential is larger reflecting the slower $j_{0,\mathrm{LFP,Li^+}}$ than $j_{0,\mathrm{g,Li^+}}$. The LFP positive electrode reaction overpotential also increases throughout the charge cycle. The electrolyte concentration overpotential is a small contribution for both cells due to the fast transport of the DME electrolytes.[3] The electrolyte and solid ohmic overpotentials are very small contributions, with the electronic conductivity in the solid domains not limiting compared to the solid ionic transport.

For the LFP vs. K-ion model the porosities were different from the TEP model, as the TEP vs. DMEe or LP57 model required higher porosities for the TEP model to run. For the LFP vs. K-ion cell the porosities were: Negative electrode = 0.25 and Positive electrode = 0.335 for both chemistries and their thicknesses adjusted to ensure NP ratio of 1.1.

Supplementary Table 3 Parameters used for PyBaMM modelling

Parameter	Value	Unit	Reference
**Cell Parameters**			
Current collector thickness	16	µm	
Current collector conductivity	3.6914×10^5	S cm^{-1}	[23]
Current collector density	2700	kg m^{-3}	[23]
Negative electrode thickness	53.319	µm	
Separator thickness	12	µm	
Positive electrode thickness	88.224	µm	
Electrode height	6.52	cm	[23]
Electrode width	1.58	m	[23]
**Negative Electrode Parameters**			
Electronic Conductivity	2.15	S cm^{-1}	[23]
Maximum concentration	23301	mol m^{-3}	This work
Diffusivity, $\widetilde{D}_{\mathrm{g}}$	2.32×10^{-13} (Supplementary Fig. 16)	cm^2 s^{-1}	This work
OCV	Fig. 2d	V	This work
Porosity	0.35	—	[8]
Active material volume fraction	0.65	—	
Particle radius	500	nm	
Bruggeman coefficient (electrolyte)	1.5	—	[23]
Bruggeman coefficient (electrode)	1.5	—	[23]
Electrons in reaction	1	—	
Charge-transfer coefficient, α_a	0.5	—	
Exchange current density, $j_{0,\mathrm{g}}$	Equation 3 and Fig. 2f	A cm^{-2}	This work
Density	2239	kg m^{-3}	[24]
**Positive Electrode Parameters**			
Electronic Conductivity	3.38×10^{-3}	S cm^{-1}	[6]
Maximum concentration	12875	mol m^{-3}	
Diffusivity, $\widetilde{D}_{\mathrm{KMF}}$	5.5040×10^{-14} (Supplementary Fig. 17)	cm^2 s^{-1}	This work
OCV	Fig. 3d	V	This work
Porosity	0.35	—	[8]
Active material volume fraction	0.65	—	
Particle radius	250	nm	
Bruggeman coefficient (electrolyte)	1.5	—	[23]
Bruggeman coefficient (electrode)	1.5	—	[23]
Electrons in reaction	2	—	
Charge-transfer coefficient, α_c	0.5	—	
Exchange current density, $j_{0,\mathrm{KMF}}$	Equation 3 and Fig. 3f	A cm^{-2}	This work
Density	2221	kg m^{-3}	This work

Supplementary Table 3 (Continued) Parameters used for PyBaMM modelling

**Separator Parameters**			
Porosity	0.47	—	[23]
Bruggeman coefficient (electrolyte)	1.5	—	[23]
Density	397	kg m^{-3}	[23]
**Electrolyte Parameters - KFSI TEP**			
Initial concentration	1.75 (2 molal)	mol L^{-1}	
Cation transference number, t_+^0	0.35	—	[21]
Thermodynamic factor, χ_M	6.5	—	[21]
Salt diffusivity, D	3.6×10^{-11}	m^2 s^{-1}	[21]
Ionic conductivity, κ	3.05	mS cm^{-1}	[21]
**Electrolyte Parameters - KFSI DME**			
Initial concentration	1.57 (2 molal)	mol L^{-1}	
Cation transference number, t_+^0	Supplementary Note 3	—	[3]
Thermodynamic factor, χ_M	Supplementary Note 3	—	[3]
Salt diffusivity, D	Supplementary Note 3	m^2 s^{-1}	[3]
Ionic conductivity, κ	Supplementary Note 3	mS cm^{-1}	[3]
**Model Parameters**			
Lower voltage cut-off	2.85	V	
Upper voltage cut-off	4.125	V	
Initial negative electrode concentration	22396	mol m^{-3}	
Initial positive electrode concentration	100	mol m^{-3}	
Initial temperature	293.15	K	

Supplementary References

1. Kang, S. D. & Chueh, W. C. Galvanostatic Intermittent Titration Technique Reinvented: Part I. A Critical Review. *J. Electrochem. Soc.* **168,** 120504 (2021).

2. O'Regan, K., Brosa Planella, F., Widanage, W. D. & Kendrick, E. Thermal-electrochemical parameters of a high energy lithium-ion cylindrical battery. *Electrochim. Acta* **425,** 140700 (2022).

3. Dhir, S., Jagger, B., Maguire, A. & Pasta, M. Fundamental investigations on the ionic transport and thermodynamic properties of non-aqueous potassium-ion electrolytes. *Nat. Commun.* **14,** 3833 (2023).

4. Landesfeind, J. & Gasteiger, H. A. Temperature and Concentration Dependence of the Ionic Transport Properties of Lithium-Ion Battery Electrolytes. *J. Electrochem. Soc.* **166,** A3079–A3097 (2019).

5. Sulzer, V., Marquis, S. G., Timms, R., Robinson, M. & Chapman, S. J. Python Battery Mathematical Modelling (PyBaMM). *J. Open Res. Softw.* **9,** 14 (2021).
6. Prada, E. *et al.* A Simplified Electrochemical and Thermal Aging Model of $LiFePO_4$-Graphite Li-ion Batteries: Power and Capacity Fade Simulations. *J. Electrochem. Soc.* **160,** A616–A628 (2013).
7. Kashkooli, A. G. *et al.* Representative volume element model of lithium-ion battery electrodes based on X-ray nano-tomography. *J. Appl. Electrochem.* **47,** 281–293 (2017).
8. Nelson, P. A., Ahmed, S., Gallagher, K. G. & Dees, D. W. *Modeling the Performance and Cost of Lithium-Ion Batteries for Electric-Drive Vehicles, Third Edition* `https://doi.org/10.2172/1503280` (2019).
9. Dhir, S., Wheeler, S., Capone, I. & Pasta, M. Outlook on K-Ion Batteries. *Chem* **6,** 2442–2460 (2020).
10. Hurlbutt, K., Wheeler, S., Capone, I. & Pasta, M. Prussian Blue Analogs as Battery Materials. *Joule* **2,** 1950–1960 (2018).
11. Wen, C. J., Boukamp, B. A., Huggins, R. A. & Weppner, W. Thermodynamic and mass transport properties of "LiAl". *Mater. Res. Bull.* **15,** 1225–1234 (1979).
12. Wang, A. A. *et al.* Review of parameterisation and a novel database (LiionDB) for continuum Li-ion battery models. *Prog. Energy* **4,** 032004 (2022).
13. Markevich, E., Levi, M. D. & Aurbach, D. Comparison between potentiostatic and galvanostatic intermittent titration techniques for determination of chemical diffusion coefficients in ion-insertion electrodes. *J. Electroanal. Chem.* **580,** 231–237 (2005).
14. Han, B. C., Van Der Ven, A., Morgan, D. & Ceder, G. Electrochemical modeling of intercalation processes with phase field models. *Electrochim. Acta* **49,** 4691–4699 (2004).
15. Coelho, A. A. *TOPAS-Academic, V6* Coelho Software: Brisbane (2016).
16. Stokes H. T. Hatch, D. M. & Campbell, B. J. *ISODISTORT,ISOTROPY Software Suite*
17. Campbell, B. J., Stokes, H. T., Tanner, D. E. & Hatch, D. M. ISODISPLACE: A web-based tool for exploring structural distortions. *J. Appl. Crystallogr.* **39,** 607–614 (2006).
18. Cattermull, J., Pasta, M. & Goodwin, A. L. Structural complexity in Prussian blue analogues. *Mater. Horiz.* **8,** 3178–3186 (2021).
19. Stephens, P. W. Phenomenological model of anisotropic peak broadening in powder diffraction. *J. Appl. Crystallogr.* **32,** 281–289 (1999).
20. Hofmann, U. & Wilm, D. Ueber die Kristallstruktur von Kohlenstoff. *Z. Elektrochem.* **42,** 504–522 (1936).
21. Zhao, J. *et al.* Transport and Thermodynamic Properties of KFSI in TEP by Operando Raman Gradient Analysis. *ACS Energy Lett.* **9,** 1537–1544 (2024).

22. Tomaszewska, A. *et al.* Lithium-ion battery fast charging: A review. *eTransportation* **1,** 100011 (2019).

23. Chen, C.-H. *et al.* Development of Experimental Techniques for Parameterization of Multi-scale Lithium-ion Battery Models. *J. Electrochem. Soc.* **167,** 080534 (2020).

24. SEC Carbon. *Fine Powder* `https://sec-carbon.com/eng/fine_powder.html`. 2012.
